# Bavachin inhibits cell viability and migration in human pancreatic cancer cells

**DOI:** 10.17912/micropub.biology.002365

**Published:** 2026-08-22

**Authors:** Caleb T Cross, Rentse GJ De Jong, Sunny P Horner, Jordan L Johnston, Cole D Davidson

**Affiliations:** 1 Biology, Wartburg College, Waverly, IA, United States

## Abstract

Pancreatic cancer is an aggressive malignancy with poor survival and limited treatment options, highlighting the need for novel therapies. Bavachin, a naturally occurring compound derived from *Psoralea corylifolia* , has demonstrated anticancer activity in other cancer models. We evaluated the effects of bavachin on PANC-1 pancreatic cancer cells *in vitro* . Sulforhodamine B and trypan blue assays assessed cell viability and determined the IC₅₀, while a wound healing assay evaluated migration. Bavachin significantly reduced PANC-1 cell viability (IC₅₀ = 30.30 μM) and inhibited migration, providing evidence that bavachin affects viability and ability to migrate in PANC-1 cells.

**Figure 1. Bavachin reduces cell viability and migration in PANC-1 cells:** (A) Bavachin decreased PANC-1 cell viability in a concentration-dependent manner following 72 hours of incubation and as measured by the SRB assay. (B) The SRB assay data were used to calculate the IC _50_ value: 30.30 µM. (C) Representative phase-contrast images illustrating cellular morphology and cell density following treatment with bavachin (Top left: 0 µM, Top right: 35 µM, Bottom left: 50 µM, Bottom right: 200 µM). (D) Bavachin (30 µM, 72 hours), corresponding approximately to the calculated IC _50_ , increased the proportion of dead PANC-1 cells compared to DMSO-treated control cells using the trypan blue assay. (E) Representative images of the migration assay (Top left: DMSO, 0 hours; Bottom left: DMSO, 48 hours; Top right: 30 µM bavachin, 0 hours; Bottom right: 30 µM bavachin, 48 hours). (F) Quantification of wound closure between treated and untreated replicates. All images were taken at 40X total magnification. Scale bar = 0.5 mm. Significance between multiple groups was determined by one-way ANOVA followed by Dunnett’s multiple comparison test where ns represents no significance (p ≥ 0.05), # represents p < 0.01, and * represents p < 0.0001. Significance between two groups was determined by a two-tailed, unpaired Student's t-test, and **** represents p < 0.0001. Error bars represent standard deviation. Each experiment was performed with three independent biological replicates.

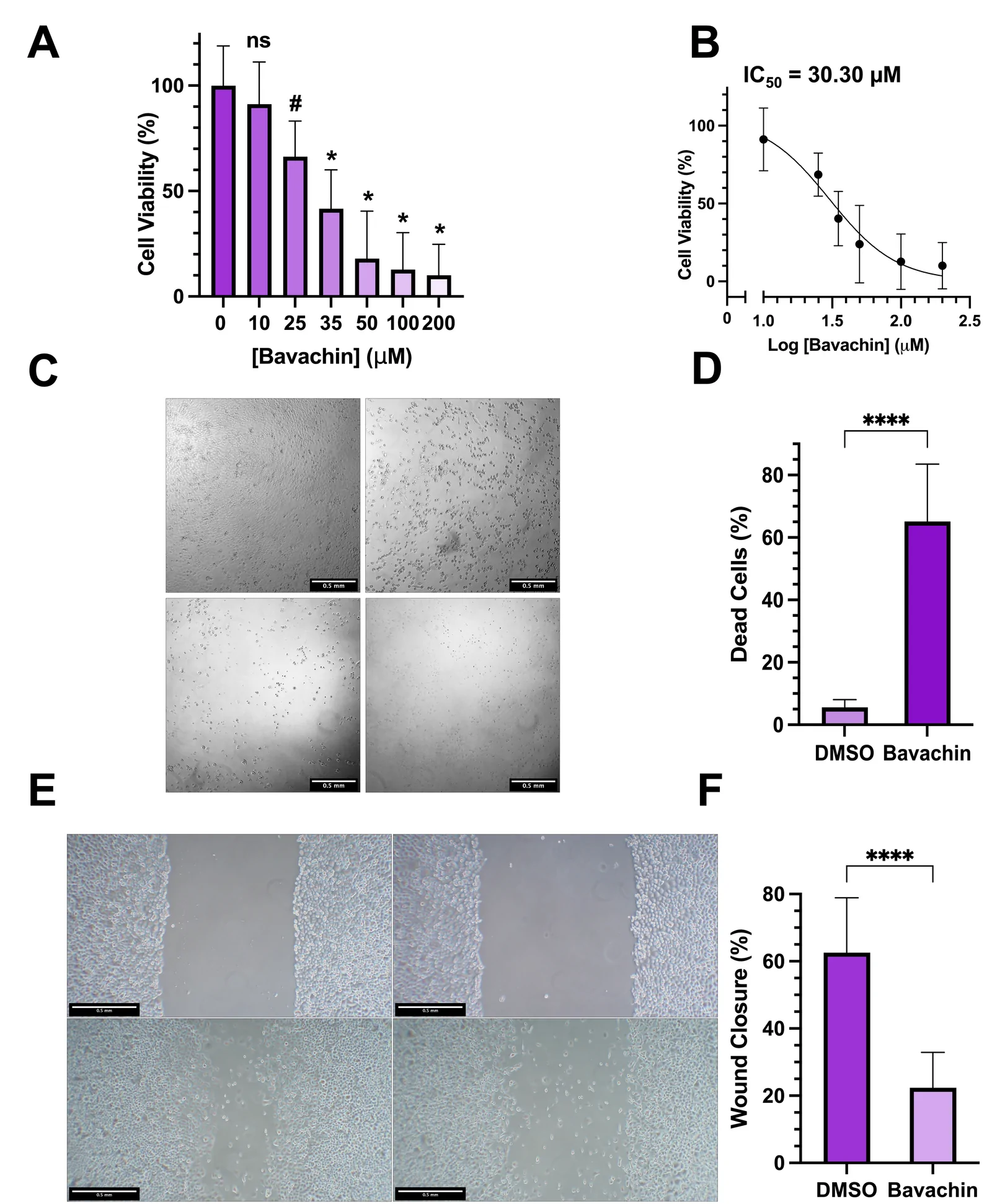



## Description

Pancreatic cancer is an aggressive and lethal malignancy with a five-year survival rate of 10% (Yang et al., 2021). It has an incidence rate of 12.5 per 100,000 in the United States but a disproportionately high mortality, with an average annual death rate of 10.9 per 100,000 patients (Zhang et al., 2018).By 2030, pancreatic cancer is projected to become the second leading cause of cancer-related mortality, surpassing breast, prostate, and colorectal cancer (Yang et al., 2021). The prognosis associated with pancreatic cancer is largely attributed to challenges in early detection, including the absence of symptoms during the initial stages and the lack of effective screening methods (Beutel & Halbrook, 2022). As a result, most patients are diagnosed after the cancer has metastasized, with only 9.7% of cases being local at the time of diagnosis (Zhang et al., 2018). Currently, no biomarker has sufficient sensitivity and specificity for routine clinical screening (Shin & Canto, 2012). Together, these factors contribute to the poor survival rate observed in pancreatic cancer patients, highlighting the pressing need for the development of effective therapeutic strategies.

Despite advances in clinical management, treatment options for pancreatic cancer remain limited. Surgical resection is the only potentially curative treatment for pancreatic cancer; however, only 15-20% of patients are eligible for surgery at diagnosis (Beutel & Halbrook, 2022). This eligibility is largely due to the advanced stage of disease at presentation, as pancreatic tumors frequently invade abdominal blood vessels and adjacent organs (Oberstein & Olive, 2013). Furthermore, recurrence rates remain high even following tumor resection. Consequently, therapies such as chemotherapy and radiation therapy are commonly used to reduce the risk of disease recurrence.

One first-line chemotherapy for pancreatic cancer is gemcitabine, a nucleoside analog of deoxycytidine (Yang et al., 2021). Gemcitabine is used in clinical oncology and has shown efficacy against cancers such as breast, ovarian, bladder, and non-small lung cancer (Beutel & Halbrook, 2022). Although gemcitabine is a therapy for pancreatic cancer, its efficacy is limited due to chemoresistance through intracellular mechanisms. Reduced expression of human equilibrative nucleoside transporter 1 (hENT1) limits drug uptake, while decreased activity of deoxycytidine kinase (dCK) reduces the conversion of gemcitabine into its phosphorylated active form (Gu et al., 2021). Additionally, increased expression of enzymes such as cytidine deaminase (CDA) metabolizes gemcitabine, thereby reducing its activity (Gu et al., 2021). Pancreatic cancer cells also exhibit extrinsic mechanisms of resistance due to the surrounding dense stroma, which acts as a biophysical barrier, segregating tumor epithelial cells and chemotherapy drugs like gemcitabine (Yang et al., 2021). Together, these challenges highlight the need to develop more effective therapeutic approaches for pancreatic cancer.

Plant-derived compounds have historically played an important role in drug discovery. More than 60% of anticancer drugs originate from natural products or their derivatives (Asma et al., 2022). Their structural diversity allows them to target multiple pathways involved in tumor growth, apoptosis, angiogenesis, and metastasis (Huang et al., 2021). Accordingly, plant-derived compounds continue to be investigated as potential therapeutic alternatives for cancers with poor treatment effectiveness such as pancreatic cancer.

One such compound that has demonstrated promising anticancer activity is bavachin. Bavachin is a flavonoid extracted from *Psoralea corylifolia* , a plant native to India and China. The seeds of this plant have traditionally been used to treat skin conditions such as eczema, psoriasis, and vitiligo (Abdullah et al., 2025). Recent studies have shown that bavachin can induce apoptosis, a form of programmed cell death, which eliminates damaged and abnormal cells (Ashing et al., 2024; Wang et al., 2023; Yang et al., 2025). Because cancer cells often escape apoptosis, restoring this process has become an important strategy for inhibiting tumor growth and improving therapeutic outcomes. Bavachin has been reported to induce apoptosis in several cancer cell types, including colorectal cancer and glioblastoma (Ashing et al., 2024; Wang et al., 2023; Yang et al., 2025). Despite these promising findings, its effects on pancreatic cancer cell viability and migration remain undocumented. Therefore, the purpose of this study was to investigate the effects of bavachin on pancreatic cancer cell viability and migration *in vitro* . We hypothesized that bavachin would reduce PANC-1 cell viability and inhibit cell migration.

To determine the effect of bavachin on pancreatic cancer cell viability, PANC-1 cells were treated with increasing concentrations of bavachin (0-200 μM ). After 72 hours, cell viability and cell death were assessed using the sulforhodamine B (SRB) and trypan blue assays. Bavachin treatment reduced PANC-1 cell viability in a concentration-dependent manner (A). No significant reduction in cell viability was observed following treatment with 10 μM bavachin (p > 0.05). Treatment with 25 μM bavachin produced a reduction in cell viability (p < 0.01), while treatment with 35, 50, 100, and 200 μM bavachin resulted in progressively greater reductions in viability (p < 0.0001). From these results, the IC _50_ of bavachin was calculated to be 30.30 μM (95% confidence interval: 16.53–37.79 μM) (B).

Representative phase-contrast images were consistent with the concentration-dependent reduction in cell number observed in the SRB assay (C). Compared with DMSO-treated control cells, cultures treated with 35 or 50 μM bavachin contained fewer and smaller cells. Furthermore, cultures treated with 200 μM bavachin contained markedly fewer adherent cells.

To independently confirm the loss of viable cells observed in the SRB assay, cell death was assessed using trypan blue exclusion at 30 µM bavachin, approximately the IC _50_ concentration. Cells treated with 30 µM bavachin showed an increase in cell death compared to the DMSO control, with 65% of cells counted as trypan blue-positive following treatment (D). These results provide additional evidence that bavachin induces cell death in PANC-1 cells (p < 0.0001).

A wound healing assay was performed to evaluate the effect of bavachin on cell migration. Compared with DMSO-treated controls, PANC-1 cells treated with 30 µM bavachin exhibited significantly reduced wound closure after 48 hours (E). Quantification of wound closure demonstrated that bavachin treatment reduced wound closure compared with DMSO-treated cells (p < 0.0001) (F).

The present findings are consistent with previous studies demonstrating the anticancer effects of bavachin in multiple cancer types. Bavachin significantly reduced the viability of PANC-1 cells, with an IC _50_ value of 30.3 µM. Similarly, Ashing et al. (2024) reported that bavachin suppressed the growth of U-87 glioblastoma cells with a slightly higher IC _50_ value of 46.00 µM. Lee et al. (2025) reported IC _50_ values of 12.1 µM and 13.6 µM in ES2 and OV90 ovarian cancer cells, respectively. These findings suggest that ovarian cancer cells may be more sensitive to bavachin than PANC-1 cells under the experimental conditions used. Luo et al. (2021) found IC _50_ values of 42.3 µM and 34.2 µM in MG63 and HOS osteosarcoma cells. Together, these studies demonstrate that bavachin has been reported to reduce viability across several cancer cell models.

In addition to inhibiting cell viability, bavachin inhibited PANC-1 migration. Bavachin-treated PANC-1 cells displayed a nearly threefold decrease in ability to close the wound compared to DMSO-treated control cells. Our findings are consistent with other studies which demonstrated an anti-migratory effect following bavachin treatment tested across multiple cancer cell lines. Yang et al. (2025) reported a significant suppression in laryngopharyngeal cancer cell migration following 20 µM bavachin treatment. Also, a recent study performed by Hsu et al. (2026) demonstrated the ability of bavachin to inhibit the migratory capacity of oral squamous cell carcinoma following treatment of 20 and 40 µM of bavachin, shown by both wound healing and transwell migration assays.

Molecular mechanisms responsible for the inhibition of PANC-1 cell migration were not specifically investigated in our study, however, a previous study using ovarian cancer cells found that bavachin inhibits ERK and p38 signaling pathways (Lee et al., 2025). Both pathways are responsible for the regulation of cytoskeletal remodeling, cell motility, and metastatic progression, which are essential for cell survival. Other molecular mechanisms were noted in a study from Luo et al. (2021), which found that bavachin induced ferroptosis in osteosarcoma cells through modulation of STAT3, p53, and SLC7A11 signaling. These previously reported effects of bavachin on cellular signaling provide potential mechanisms that may contribute to the effects observed in PANC-1 cells.

While bavachin demonstrated significant cytotoxic and anti-migratory effects in PANC-1 cells, all experiments were performed *in vitro* . Future studies should evaluate bavachin in more physiologically relevant models, such as organoid systems and *in vivo* models to determine whether its therapeutic effects are maintained in the presence of complex tumor environments, characteristic of pancreatic cancer. Furthermore, the pathways responsible for the observed reduction in PANC-1 cell viability and migration were not investigated. Future studies should identify the molecular mechanisms responsible for bavachin-induced reductions in cell viability and migration in accordance with previous studies in ovarian and osteosarcoma cells (Lee et al., 2025; Luo et al., 2021). Finally, the present study did not evaluate the effects of bavachin in non-cancerous cells; therefore, the selectivity of bavachin for pancreatic cancer cells relative to non-cancerous cells remains unknown.

In conclusion, our hypothesis was supported, as bavachin reduced PANC-1 cell viability, increased the proportion of trypan blue-positive cells, and reduced wound closure *in vitro* . These findings provide evidence that bavachin affects viability and wound closure in PANC-1 cells and warrant further investigation in more physiologically relevant models.

## Methods

**Cell Culture**

Human pancreatic ductal adenocarcinoma (PANC-1) cells (American Type Culture Collection, Manassas, VA, USA) were cultured in T-75 flasks with 10 mL of Eagle's Minimum Essential Medium (EMEM; Corning, Corning, NY, USA) supplemented with 10% fetal bovine serum (FBS), penicillin (100 IU/L) (Thermo Fisher Scientific, Waltham MA, USA), and streptomycin (100 μg/mL) (Corning). Cells were maintained at 37 °C in a humidified incubator with 5% CO _2_ . Upon reaching approximately 70% confluence, media were aspirated, cells were washed with 10 mL phosphate-buffered saline (PBS) (Thermo Fisher), and cells were detached using 2.1 mL 0.25% trypsin (Thermo Fisher) for 5 minutes at 37 °C. Cells were collected with 8 mL media to neutralize the trypsin, and cell counts were determined using a hemocytometer prior to each experiment. All experiments used PANC-1 cells that were at most 5 passages old from thawing.

**Sulforhodamine B (SRB) Assay**

Cell viability was assessed using the SRB assay previously described (Leek et al., 2026). Briefly, PANC-1 cells were seeded in 96-well plates at a density of 5,000 cells per well and allowed to adhere overnight. Cells were treated with increasing concentrations of bavachin or an equivalent volume of DMSO vehicle control for 72 hours. Cells were then fixed with trichloroacetic acid, stained with SRB, washed with acetic acid, and the bound dye was solubilized with tris base. Absorbance was measured at 564 nm using a SpectraMax 190 Microplate Reader (Molecular Devices, San Jose, CA, USA), and values were normalized to the DMSO-treated control. Representative phase-contrast images were acquired at 40X magnification using an Olympus BX41 microscope equipped with an AmScope HD202-MW camera. 

**Trypan Blue Assay**

A trypan blue exclusion assay was performed as an independent assay to confirm the loss of viable cells observed in the SRB assay following bavachin treatment. PANC-1 cells were seeded into six-well plates (Advangene Consumables, Lake Bluff, IL, USA) at a density of 1.0 x 10 ^5^ cells per well with 2 mL media per well. Cells were allowed to adhere overnight before treatment. PANC-1 cells were treated with 30 µM bavachin, approximately the concentration corresponding to the calculated IC _50_ , or an equivalent volume of DMSO vehicle control. After 72 hours of incubation, media were aspirated, and cells were rinsed with 2 mL of PBS per well. Cells were lifted using 200 μL of 0.25% trypsin per well and incubated for five minutes at 37 °C. Then, cell suspensions were diluted 1:1 in 0.4% (w/v in PBS) trypan blue solution (Sigma-Aldrich). Cell suspensions were loaded onto a hemocytometer, and viable (unstained) and nonviable (blue-stained) cells were manually counted. The percentage of dead cells was calculated by dividing the number of nonviable cells by the total number of cells counted.

**Cell Migration Assay**

The cell migration assay was conducted as described previously (Boge et al., 2026). Briefly, 1.0 X 10 ^6^ PANC-1 cells were seeded into 6-well culture plates and allowed to reach near-complete confluency overnight. The media were aspirated from each well, and a vertical scratch (wound) was created through the cells using a sterile 200-μL pipette tip. Detached cells were removed by washing the wells with 2 mL of warm PBS. Bavachin (30 μM) or DMSO vehicle control were added to each well in media containing 2% fetal bovine serum to limit proliferation. Images of the wound area were captured immediately following scratch formation (0 hours) and after 48 hours of incubation. Cell migration was quantified by measuring wound closure over time using ImageJ software version 1.53k (Schneider et al., 2012; Suarez-Arnedo et al., 2020). The migration assay was performed in three independent biological replicates with each biological replicate consisting of three technical replicates per treatment group.

**Data Analysis**

All statistical analyses were performed using GraphPad Prism version 11.0.0 (GraphPad Software, Boston, MA, USA). Normality of the data was assessed using the Shapiro-Wilk test. Comparisons among multiple groups were performed using one-way ANOVA followed by Dunnett's multiple comparison test; ns represents no significance (p ≥ 0.05), # represents p < 0.01, and * represents p < 0.0001. Significance between two groups was determined by a two-tailed, unpaired Student's t-test, where **** represents p < 0.0001. Data are presented as mean ± standard deviation from three independent biological replicates per group. 
